# The Activities of Recombinant Botulinum Toxin A on Spared Nerve Injury-Induced Neuropathic Pain in a Diabetic Mice Model

**DOI:** 10.3390/toxins17110545

**Published:** 2025-11-03

**Authors:** Akinyemi Ademola Omoniyi, Rasmus Eich Hammer, Sabrina Josefsen, Mette Richner, Stephane Lezmi, Christian Bjerggaard Vægter, Mikhail Kalinichev, Páll Karlsson, Jens Randel Nyengaard

**Affiliations:** 1 Core Center for Molecular Morphology, Section for Stereology and Microscopy, Department of Clinical Medicine, Aarhus University, 8200 Aarhus N, Denmark; aaomoniyi@abu.edu.ng (A.A.O.); rasmuseichhammer@gmail.com (R.E.H.); sabrina.stobberup.josefsen@regionh.dk (S.J.); pall@clin.au.dk (P.K.); 2 Department of Human Anatomy, Ahmadu Bello University, Zaria 810001, Nigeria; 3 Danish Research Institute of Translational Neuroscience (DANDRITE), Nordic-EMBL Partnership for Molecular Medicine, Department of Biomedicine, Aarhus University, 8000 Aarhus C, Denmark; mette.richner@clin.au.dk (M.R.); cv@biomed.au.dk (C.B.V.); 4 Ipsen Innovation, Avenue du Canada, 91940 Les Ulis, France; stephane.lezmi@gmail.com (S.L.); mikhail.kalinichev@icloud.com (M.K.); 5 Danish Pain Research Center, Department of Clinical Medicine, Aarhus University, 8000 Aarhus C, Denmark; 6 Department of Pathology, Aarhus University Hospital, 8200 Aarhus N, Denmark

**Keywords:** peripheral nervous system, von Frey, Iba1, GFAP, microglia, astrocytes, diabetic neuropathy

## Abstract

Diabetic neuropathy is characterized by nerve damage and chronic neuropathic pain and lacks effective treatment. Botulinum neurotoxin type A (BoNT/A), a neurotoxin with established therapeutic use in neurological disorders, has emerged as a potential analgesic agent. This study investigated the effects of a recombinant form of BoNT/A1 (rBoNT/A1) on neuropathic pain induced by spared nerve injury (SNI) in a diabetic mouse model. Thirty-two adult male C57BL/6JRj diabetic mice were subjected to SNI or sham surgery. Fourteen days post surgery, mice received an intraplantar dose of rBoNT/A1 or vehicle. Mechanical allodynia was assessed using von Frey filaments, and spinal cord and sciatic nerve tissues were analyzed via immunohistochemistry and transmission electron microscopy to evaluate glial activation, neurotransmitter receptor expression, and axonal morphology. The results demonstrated that rBoNT/A1 significantly alleviated mechanical allodynia and caused a marked reduction in Iba1-positive microglial activation in the spinal cord, whereas no significant changes were observed in astrocyte (GFAP) density or GABAAR subunit expression. Additionally, rBoNT/A1 treatment did not significantly alter axon diameter, myelin thickness, or C-fiber morphology. In conclusion, intraplantar administration of rBoNT/A1 reduced SNI-induced mechanical allodynia in diabetic mice, potentially by attenuating spinal microglial activation, supporting the therapeutic promise of rBoNT/A1 in managing diabetic neuropathic pain.

## 1. Introduction

Diabetes mellitus is a widespread metabolic disorder that imposes a significant burden on global healthcare systems, with one of its primary complications being diabetic neuropathy [1]. This multifaceted condition, characterized by nerve damage due to prolonged hyperglycemia, leads to neuropathic pain, severely affecting the quality of life for those affected [2]. The intricate interplay of metabolic, vascular, and neural factors contributes to the development and persistence of neuropathic pain in diabetes [3]. Diabetic polyneuropathy, a specific form of diabetic neuropathy, is defined as peripheral nerve dysfunction [4], most often associated with type 2 diabetes. Chronic hyperglycemia in diabetic polyneuropathy induces inflammation, oxidative stress, and mitochondrial dysfunction, leading to the production of reactive oxygen species (ROS) and subsequent nerve damage, including segmental demyelination and axonal degeneration [5]. The condition is often accompanied by severe pain, thought to result from altered synaptic activity in neurons following nerve damage [6]. Neuronal hyperactivity contributes to the perception of non-painful stimuli as painful (allodynia) and amplifies painful stimuli (hyperalgesia) [4]. Recent studies suggest that interactions between neurons and glial cells may play a role in contributing to neuropathic pain associated with diabetic polyneuropathy [7,8,9].

Spared nerve injury (SNI) serves as a well-established animal model for studying neuropathic pain, simulating the clinical scenario by inducing focal nerve damage and subsequent pain behavior [10]. The relevance of SNI in diabetic neuropathy research lies in its ability to recapitulate key aspects of neuropathic pain, including mechanical allodynia and thermal hyperalgesia, observed in diabetic patients [10,11]. Animal models of diabetes, particularly those based on streptozotocin (STZ)-induced type 2 diabetic neuropathy, are well established for studying the underlying mechanisms of neuropathic pain without requiring an additional injury. However, combining diabetic conditions with a peripheral nerve injury model, such as spared nerve injury (SNI), provides a more complex and clinically relevant paradigm that better mimics the multifactorial nature of neuropathic pain seen in diabetic patients. This approach allows for the investigation of interactions between metabolic dysfunction and nerve trauma within a controlled experimental framework.

Approximately 8% of the general population suffers from neuropathic pain, and while diabetes is accountable for approximately 30% of these cases, there exist hundreds of other conditions that are associated with neuropathic pain [12]. Currently, pharmacological treatments include non-steroidal anti-inflammatory drugs (NSAIDs), antidepressants, anticonvulsants, and opioids. However, treatment often involves prolonged usage of these medications; thus, adverse effects and reduced analgesic efficacy are typical problems [6]. Recently, botulinum neurotoxin A (BoNT/A) has attracted attention for its potential analgesic properties in various pain models. While BoNT/A has shown promising preclinical and clinical results, its approved therapeutic use for pain is currently limited to certain conditions under the BOTOX^®^ formulation. Therefore, its broader use as an analgesic remains investigational and should be interpreted with scientific caution.

Botulinum Toxin A (BoNT/A), derived from Clostridium botulinum, is a potent neurotoxin that has been approved as a therapeutic option for various neurological conditions, including chronic pain syndromes [13]. Its mechanism of action involves inhibiting neurotransmitter release, particularly acetylcholine, at the neuromuscular junction, leading to the alleviation of conditions like muscle spasms, dystonia, and chronic pain [14]. Recombinant forms of BoNT/A (rBoNT/A) have been developed to enhance purity and consistency compared to native counterparts [15].

Despite its inherent toxicity, BoNT/A is increasingly used as a therapeutic agent [6,16] in clinical settings to reduce pain associated with conditions such as migraines and arthritis [16]. The exact mechanism by which BoNT/A alleviates pain requires further investigation. BoNT/A inhibits neurotransmitter release by cleaving SNAP-25, resulting in a dysfunctional SNARE complex that prevents synaptic vesicles from fusing to the nerve terminal membrane [16]. In addition to inhibiting acetylcholine release in neuromuscular junctions, BoNT/A also affects the exocytosis of local nociceptive peptides involved in nociceptive transmission, including substance P [17], calcitonin gene-related peptide (CGRP) [18], and glutamate [19]. While BoNT/A is considered potentially effective in treating neuropathic pain, most evidence comes from small in vivo and in vitro studies [20,21]. Only a limited number of clinical studies have explored the use of intradermal BoNT/A injections for neuropathic pain associated with diabetic neuropathy, revealing reduced pain reported by patients [22,23]. However, the mechanisms underlying the toxin’s anti-allogenic effect in diabetic neuropathy remain understudied, prompting the need for further in vitro investigations.

While the application of BoNT/A in neuropathic pain management has shown promise, its specific impact on neuropathic pain in the context of diabetic mice models with SNI remains an understudied area. Therefore, this study aims to elucidate the activities of rBoNT/A in ameliorating SNI-induced neuropathic pain in diabetic mice. By addressing this research gap, we strive to contribute valuable insights into the potential therapeutic avenues for managing neuropathic pain in individuals with diabetes.

## 2. Results

### 2.1. Body Weight

**Figure 1:**
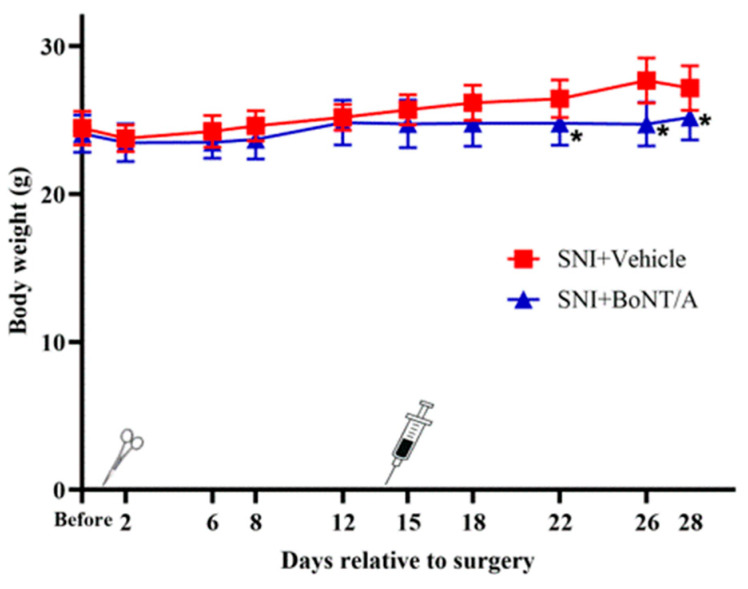
Body weight of the diabetic mice over the period of the experiment. Independent *t*-test, *p* < 0.05. Mean ± SD. *n* = 8. Scissors = intraplantar injection of rBoNT/A1 or vehicle on day 14 post operation, syringe = spared nerve injury operation. * = significantly different when compared to it pair.

All mice showed an increase in mean body weight throughout the entire experiment, regardless of treatment group. Before day 21 (7 days post injection), there was no statistically significant difference in weight gain between the SNI+rBoNT/A1 and SNI+Vehicle groups. After day 21, rBoNT/A1-injected mice had a significantly lower body weight compared to vehicle-injected mice; thus, weight gain was lower for rBoNT/A1-injected mice in this time period (Figure 1). The result of Cohen’s *d* computation was 1.21, indicating that the intervention had a large effect on the weight of the mice at day 8 post injection.

### 2.2. Von Frey Measurements

**Figure 2:**
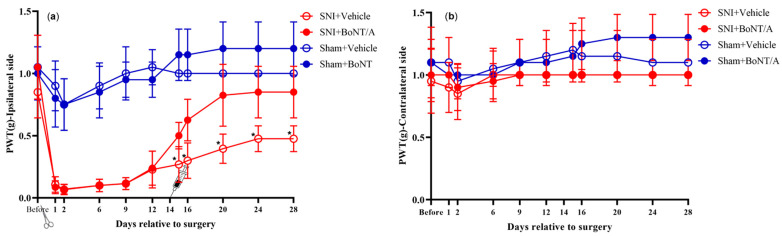
von Frey measurements of paw withdrawal threshold (PWT) for the treatment groups in normal mice: (**a**) ipsilateral hind limb, between (*p* < 0.001) and within (*p* < 0.001) the treatment group effect; (**b**): contralateral hind limb, between (*p* = 0.001) and within (*p* = 0.008) the treatment group effect. Mixed-design ANOVA. Mean ± SD. *n* = 8. Scissors = intraplantar injection of rBoNT/A1 or vehicle on day 14 post operation, syringe = spared nerve injury operation. * = significantly different when compared to its pair.

Paw withdrawal threshold (PWT) is a measure of mechanical allodynia (Figure 2). When considering the PWT on the ipsilateral side across all four treatment groups, a mixed-design ANOVA revealed that the interaction between treatment group and time had a significant effect (*p* < 0.05). The results indicated that the neuropathic surgery significantly (*p* < 0.001) decreased the paw withdrawal threshold (PWT) in mice with the partial eta squared (*η*^2^) of 0.68.

The injection of rBoNT/A1 on day 14 following the SNI significantly increased the PWT with *η*^2^ of 0.91, indicating that the injection of rBoNT/A1 has a large effect in relieving mechanical allodynia following SNI. Following injection, the PWT of SNI+vehicle mice (0.27 ± 0.05 g) was significantly lower compared to SNI+rBoNT/A1 (0.50 ± 0.03 g), sham+vehicle (1.0 ± 0.0 g), and sham+rBoNT/A1 (1.15 ± 0.07 g). These treads remained consistent to the end of the experiment (i.e., day 28 post operation). Based on conventional guidelines [24] on effect size, these observed effects were large.

### 2.3. Number Density of Iba1-Marked Cells in the Spinal Cord

**Figure 3:**
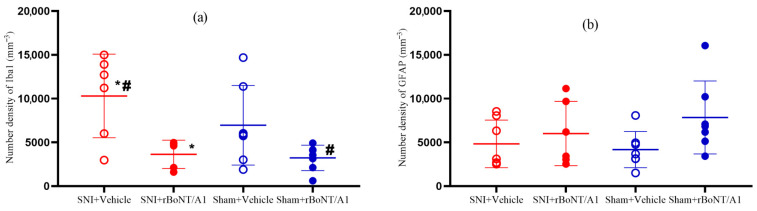
Comparison between the four treatment groups of diabetic mice: (**a**) mean number density of Iba1-marked microglia (*p* = 0.007); (**b**) mean number density of GFAP-marked astrocytes (*p* = 0.191). Experimental groups with * or # are significantly different from group labelled with *#. One-way ANOVA, post hoc: LSD. Mean ± SD. *n* = 8. rBoNT/A1 injection: 14 days post nerve injury period. Sample collection: 12 days post nerve injury period.

Among the four treatment groups, there was a statistically significant (*p* = 0.007, *η*^2^ = 0.43) reduction in the number density of Iba1-marked microglia in the spinal cords of rBoNT/A1-injected mice compared to vehicle-injected mice (Figure 3a). The *η*^2^ effect size was 0.43, indicating that rBoNT/A1 or vehicle injection had a large effect on Iba1-marked microglia in the spinal cord [24]. Based on the effect size value, 43% of the variance in Iba1-marked microglia was accounted for by rBoNT/A1.

#### 2.3.1. Number Density of GFAP-Marked Cells in the Spinal Cord

The one-way analysis of variance test was not statistically significant (*p* = 0.191, *η*^2^ = 0.18) in the number density of GFAP-marked cells. The eta squared effect size was 0.18, indicating that rBoNT/A1 or vehicle injection had a medium effect on the number density of GFAP-marked cells. Based on the effect size value, 18% of the variance in the number density of GFAP-marked cells was accounted for by rBoNT/A1 or vehicle injection. See Figure 3b.

#### 2.3.2. GABA_A_R Subunits in the Spinal Cord

**Figure 4:**
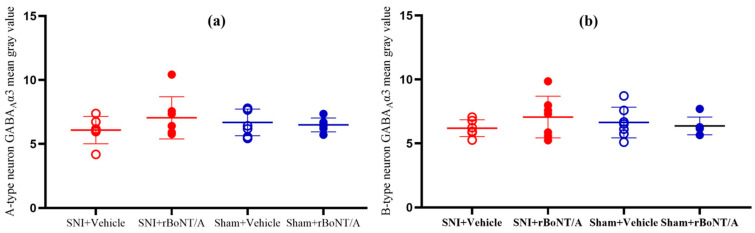
Comparison between the four treatment groups in diabetic mice: (**a**) mean gray value of GABAA α3 receptor in A-type DRG neuron (*p* =0.191); (**b**) mean gray value of GABAA α3 receptor in B-type DRG neuron (*p* = 0.139). One-way ANOVA. Mean ± SD. *n* = 8. rBoNT/A1 injection: 14 days post nerve injury period. Sample collection: 14 days post injection period.

The four treatment groups showed no difference in fluorescence intensity (aCTCF) or mean fold change (MFC) of GABA_A_R subunits. See Figure 4.

#### 2.3.3. Axon Area, C-Fiber Diameter, and Myelin Thickness

**Figure 5:**
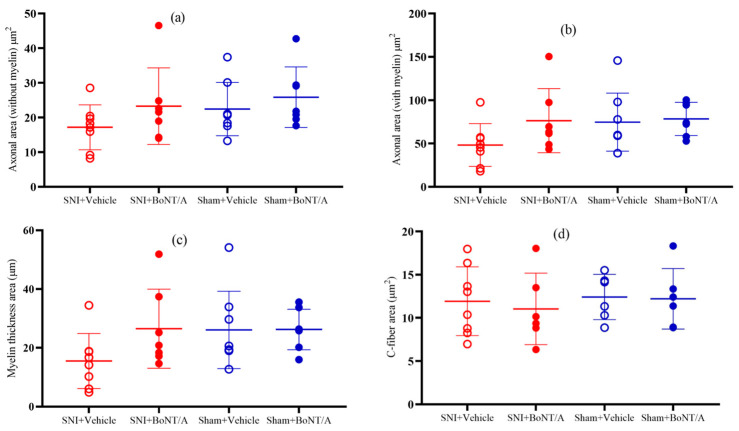
Comparison between the four treatment groups in diabetic mice: (**a**) axon area without myelin (*p* = 0.269); (**b**) axon area with myelin (*p* = 0.171); (**c**) area of the myelin thickness (*p* = 0.165); (**d**) C-fiber area (*p* = 0.918). One-way ANOVA. Mean ± SD. *n* = 8. rBoNT/A1 injection: 14 days post nerve injury period. Sample collection: 14 days post injection period.

The one-way analysis of variance was not statistically significant (*p* = 0.269, *η*^2^ = 0.14) in the axon area, myelin thickness around the axon (*p* = 0.165, *η*^2^ = 0.18), and C-fiber area (*p* = 0.918, *η*^2^ = 0.02). The eta squared effect size of 0.14, 0.18, and 0.02 indicates that rBoNT/A1 or vehicle injection had a large effect on the area of axon without myelin, myelin thickness around the axon, and a small effect on the C-fibers, respectively. Based on the effect size value, 14%, 18%, and 2% of the variance in axon area, myelin thickness around the axon, and C-fiber, respectively, were accounted for by rBoNT/A1 or vehicle injection. See Figure 5.

## 3. Discussion

A variety of studies suggest that BoNT/A has an analgesic effect [20,21,25,26]; however, the underlying mechanism is still debated. Additionally, no studies have yet investigated such an effect in diabetic polyneuropathy. Correspondingly, this study used von Frey measurements to demonstrate increased pain threshold, i.e., decreased mechanical allodynia, following intraplantar injection of rBoNT/A in mice exposed to SNI, a model of neuropathic pain. Considering the propagation of such nociceptive stimuli, several markers within the spinal cord were identified as potential targets for BoNT/A. Analysis of microglia and astrocyte numbers in the spinal cord indicated microglia activation following SNI surgery, and this effect was reversed by rBoNT/A injections. No changes were apparent in astrocyte numbers, nor were levels of GABA_A_R subunits in the spinal cord affected by these interventions. Finally, myelin thickness was nearly identical in all treatment groups.

### 3.1. Von Frey Measurements

A prompt and significant reduction in ipsilateral PWT was observed in SNI-operated mice, but not in sham-operated mice. This clearly illustrates that mice developed mechanical allodynia as a result of SNI. Mechanical allodynia was sustained throughout the experiment (28 days), which parallels previous studies [27]. The anti-allodynic effect of BoNT/A has been demonstrated in both SNI models [28] and CCI models [29,30,31] of neuropathic pain. Correspondingly, this experiment showed a significant increase in ipsilateral PWT among SNI-operated mice following intraplantar rBoNT/A injection, compared to a vehicle injection. This effect was seemingly sustained for 14 days post injection (day 28); however, the dwindling allodynic effect of SNI surgery may disguise a decreasing anti-allodynic effect of BoNT/A. Previous studies have reported a sustained anti-allodynic effect of BoNT/A for 14 days regarding the SNI model [28], and for 12, 21, and 81 days regarding the CCI model [29,30,31]. However, rBoNT/A administration before nerve injury does not appear to reduce mechanical allodynia [32]. The toxin dosage administered in the above-mentioned studies was not identical; thus, direct comparison is difficult.

Previous studies have discussed the migration patterns of unilaterally injected BoNT/A, as unilateral BoNT/A administration may have bilateral anti-allogenic effects. In our study BoNT/A did not appear to affect PWT in the contralateral paw. Similarly, rats exposed to CCI and ipsilateral aboBoNT/A injection did not exhibit reversed hyperalgesia in the contralateral paw. However, a unilateral BoNT/A injection in rats with streptozotocin-induced diabetic polyneuropathy did reverse hyperalgesia bilaterally [33]. Such findings lead to the discussion of possible retrograde axonal transport of BoNT/A, as this could explain the bilateral effects of a unilateral injection [16,34]. The authors also allude to the possibility that the toxin may limit the release of pain mediators at the injection site, which in turn reduces central sensitization and thereby has bilateral anti-allogenic effects [33]. However, this requires further investigation.

The anti-allodynic effect of BoNT/A was supported by mice regaining normal posture and locomotion. Following SNI surgery, mice refrained from bearing weight on the ipsilateral paw, and they preferred to lean on the contralateral hind paw. However, these patterns disappeared after treatment with BoNT. Similar observations have been reported in previous studies [31]. Notably, in our study, the toxin did not affect mechanical thresholds in sham-operated diabetic mice but produced significant analgesia following spared nerve injury (SNI). This selective effect suggests a state-dependent mechanism targeting injury-induced neuroinflammatory pathways rather than basal nociception, consistent with previous evidence linking glial modulation to context-specific analgesia.

### 3.2. Glial Cells

Several studies considering mice and rats have shown that peripheral BoNT/A administration reverses the activation of glial cells in the spinal cord caused by a sciatic nerve injury [6,8,32]. This appears to be true for microglia, but there are discrepancies when considering astrocytes [6]. This experiment correspondingly showed a general tendency of increased microglia activation following SNI operation. After BoNT/A injections, the number of active microglia within the spinal cord was significantly reduced. This pattern was not evident for astrocytes. Research has shown that in the context of diabetes, glial cells undergo activation, leading to neuroinflammation, glutamate toxicity, and changes in synaptic transmission, which are associated with the development of diabetic neuropathy and pain. The activation of glial cells, such as microglia and astrocytes, has been linked to the release of pro-inflammatory factors and the modulation of synaptic transmission, contributing to the pathogenesis of diabetic neuropathic pain [35,36,37].

Mika et al. [8] exposed rats to a chronic constriction injury (CCI) of the sciatic nerve and observed both mechanical allodynia and thermal hyperalgesia on the ipsilateral side. These observations were accompanied by an increase in microglia cells and astrocytes in the ipsilateral side of the spinal cord, thus supporting the idea that nerve injury leads to a pro-inflammatory environment. Following intraplantar BoNT/A injections, all the effects of CCI were reversed: numbers of microglia in the ipsilateral side of the spinal cord were reduced, and the rats presented less neuropathic pain-related behaviors. Interestingly, BoNT/A injections 3 days before CCI had the same effect as BoNT/A injections 5 days after CCI. This suggests that BoNT/A may block injury-induced changes [31].

Following a nerve injury, toll-like receptors (TLR2, TLR4) on the surface of microglia will recognize danger-associated molecular patterns (DAMPs), which subsequently activate the MAPK signaling pathway (decreased phosphorylation of p38 and ERK 1/2) in microglia and lead to the release of pro-inflammatory cytokines (IL-1β, IL-18). This neuro-inflammation results in hyperactivity of sensory neurons, which causes pain [38,39]. Correspondingly, intrathecal injections of IL-1β and IL-18 in rats trigger pain-like behaviors [8]. Cell cultures of LPS-activated microglia treated with BoNT/A show inhibited LPS-induced phosphorylation of these intracellular signaling molecules, thereby reducing microglia activation and subsequent release of pro-inflammatory factors [40]. This suggests that BoNT/A is capable of reversing the pro-inflammatory imbalance caused by nerve injuries, thus reducing hyperactivity in sensory neurons and relieving pain. This effect was not seen in astrocytes, which is consistent with the many animal studies that fail to show a change in astrocyte activation in spinal cords, following intraplantar BoNT/A injections [41]. The involvement of glial cells in diabetic neuropathic pain is a subject of ongoing research, and the understanding of the specific mechanisms and potential therapeutic targets is continuously evolving. A limitation of the present study is that microglial morphology was not analyzed. Our assessment focused on total Iba1-positive microglial density, which does not distinguish between ramified (resting) and amoeboid (activated) phenotypes. Future studies incorporating morphological and phenotypic analyses will be essential to determine whether the toxin’s effect reflects reduced microglial activation or overall cell number.

### 3.3. Axon Diameter, C-Fiber Diameter, and Myelin Thickness

Peripheral neuropathies are common complications in patients with diabetes mellitus, one of which is idiopathic chronic inflammatory demyelinating polyneuropathy (I-CIDP). In this condition, a pro-inflammatory environment triggers demyelination [42]. Research has shown that diabetic individuals with polyneuropathy exhibit elevated levels of tumor necrosis factor alpha, membrane attack complex components [43], and autoantibodies against phospholipids and gangliosides, all of which contribute to the demyelination process. Immunotherapy targeting these factors is effective in some cases of diabetic neuropathy [42]. However, in the present study, BoNT/A did not significantly alter myelin thickness or axon diameter. Despite this, other research has demonstrated that BoNT/A encourages the proliferation and maturation of Schwann cells, which are essential for myelinating axons and promoting nerve regeneration, suggesting a potential role for BoNT/A in enhancing nerve repair [44].

## 4. Conclusions

This study showed that rBoNT/A injections increase the PWT of the ipsilateral paw after SNI surgery in diabetic mice, thus demonstrating that the toxin reduces mechanical allodynia and possibly neuropathic pain. The underlying mechanism requires further research; however, rBoNT/A1 appears to reverse the activation of microglia cells in the spinal cord, leading to reversal of neuroinflammation, glutamate toxicity, and changes in synaptic transmission associated with the development of diabetic neuropathy triggered by SNI surgery.

## 5. Materials

Adult male db/db mice (BKS.Cg-Dock7m+/+Leprdb/J; 10–12 weeks old, 25–35 g) were obtained from Janvier Labs (Le Genest-Saint-Isle, France). These mice carry a spontaneous mutation in the Leprdb gene and serve as a well-characterized model of type 2 diabetes mellitus, exhibiting obesity, hyperglycemia, and insulin resistance by 8–10 weeks of age. Throughout this study, mice were housed with littermates, four animals per Type III cage (Tecniplast Eurotype 1290D cage, Buguggiate, Italy 425 × 276 × 153 mm). The bedding material of Tapvei 2HV bedding and LBS soft paper wool nesting material were cleaned and replaced weekly. A week before the experiment, mice were acclimatized at 23 ± 2 °C temperature, 55 ± 5% humidity, and on a 12:12 h light/dark cycle (lights turned on between 7:00 and 19:00 h). Food and water were given ad libitum. The Danish Animal Experiment Inspectorate approved all experiments in this study (permission nr 2017-15-0201-01192; approved on 15 February 2017).

The recombinant form of BoNT/A1 (rBoNT/A1; IPN10260) was synthesized from the native amino acid sequence expressed in *Escherichia coli*, and provided by Ipsen Bioinnovation Ltd. (Milton Park, Milton, UK). Examination of rBoNT/A1 in vitro and ex vivo showed biochemical and functional similarities to native BoNT/A1 (nBoNT/A1) [15]. Additionally, recombinant- and native-BoNT/A1 were equipotent, and when applied in vivo, in mouse and rat digit abduction score (DAS) assays following a single intramuscular injection, the onset and duration of action were similar for both types of BoNT/A1 [15].

### 5.1. Study Design

**Figure 6:**
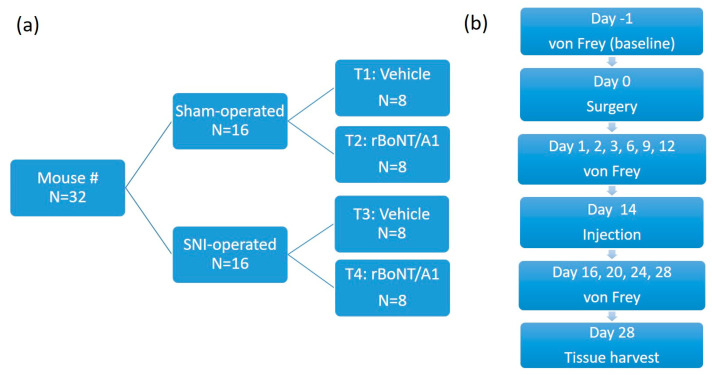
(**a**) Overview of treatment groups; (**b**) a timeline of the interventions. Vehicle = gelatine phosphate buffer (GPB). Surgery = spared nerve injury (SNI) surgery. Injection = intraplantar injection of either 3.2 pg. rBoNT/A1 or the vehicle (GPB).

A total of 32 mice were used in the main study: 16 were spared nerve injury (SNI)-operated, and 16 were sham-operated (open and closed again). On day 14 post surgery, following stabilization of the pain threshold, eight mice from each surgery group received an intraplantar BoNT/A injection at the left hind paw (the same paw in which mechanical allodynia after SNI was expected to occur). The injection consisted of either 3.2 pg. rBoNT/A (IPN10260; Ipsen Bioinnovation, Milton, UK) diluted in 20 µL gelatine phosphate buffer (GPB) or simply a vehicle injection (20 µL GPB). At 14 days post injection (now 28 days following surgery), tissue samples were collected from all 32 mice. An online research tool (randomizer.org, accessed on 3 April 2019) was used to ensure treatment allocation was random. An overview of the treatment groups is shown in Figure 6. All experiments were conducted by the same investigator, who remained blinded throughout the trials.

### 5.2. Surgery

Fourteen days before rBoNT/A or vehicle injection, the mice were operated on according to the spared nerve injury (SNI) model [10], which intends to mimic polyneuropathy. A small skin incision was made at the mid-thigh level, followed by a blunt dissection through the biceps femoris muscle to expose the sciatic nerve and its three branches. Distally to the trifurcation of the sciatic nerve, the common peroneal and tibial nerve branches were unilaterally ligated using nonabsorbable suture (polypropene 6-0), Ethicon. Hereafter, the common peroneal and tibial nerve branches were cut distally to the ligation, while the sural nerve was left intact. For the sham surgery, the sciatic nerve was exposed but neither ligated nor cut. The incisions were closed with tissue adhesive (Klin-ibond, Utrecht, The Netherlands). Mice were anesthetized with sevoflurane gas (AbbVie A/S, Hillerød, Denmark), administered via a UNO Basic Anaesthesia Set-up for small rodents, and Lidocaine SAD (10 mg/mL; AstraZeneca, Cambridge, UK) was applied topically to the wound site. Ampicillin STADA (STADA Nordic, Herlev, Denmark) was reconstituted in isotonic saline to yield a final concentration of 250 mg/mL, and Temgesic (0.3 mg/mL, INDIVIOR Limited, Hull, United Kingdom) was diluted and mixed 1:10 in isotonic saline (9 mg/mL, Fresenius Kabi, Homburg, Germany). This solution (0.1 mL) was subcutaneously injected. The mice involved in this experiment had substantial adipose tissue; thus, suture leaks after surgery were common. These mice were re-sutured.

### 5.3. Testing Mechanical Sensitivity

A standardized set of five von Frey filaments (Semmes–Weinstein monofilaments, Stoelting; Wood Dale, IL, USA), with varying thresholds, was applied in ascending order to assess mechanical sensitivity. Each filament was applied to each mouse five times, and the five filaments were tested in ascending order. Each filament was placed at a 90 °C angle to the lateral portion of the ipsilateral (operated) hind paw, and held for 30 s. During this time, the mouse’s pain perception was evaluated. A positive reaction was defined as vigorous paw withdrawal, flinching, or licking responses occurring during three or more of five stimulations. After assessing mechanical sensitivity for all mice, the same method was applied to the contralateral (non-operated) paw, starting with the mouse that was tested first on the unilateral hind paw. Baseline von Frey measurements were determined one day before SNI surgery (day 0). Mice were habituated during the light phase for at least 15 min before testing. All tests were conducted by the same researcher, to reduce sex and odor bias [45] as well as person-to-person variation.

### 5.4. Tissue Harvesting and Preparation

Following deep anesthetization and decapitation, spinal cords were harvested by hydraulic extrusion [46]. Firstly, the skin was cut along the spine, and the spinal column was separated from the surrounding tissue by cutting on both sides of the spinal column as well as distally to the pelvic bone. A syringe with an adjusted pipette tip was inserted at the caudal end of the spinal cord, and using gentle pressure, ice-cold PBS was used to extrude the spinal cord into a Petri dish on ice containing PBS. Then, the spinal column was cut longitudinally and fixed in paraformaldehyde (4% PFA). The lumbar enlargement of each spinal cord was cut into three equal blocks. Spinal cords were immersion-fixed in 4% phosphate-buffered formaldehyde, dehydrated using increasing concentrations of ethanol, and finally xylene. These blocks were then embedded in paraffin. The resulting paraffin blocks were cut into parallel series of 3 μm sections using an automated rotary microtome (RM2255, Leica, Wetzlar, Germany). Two series, each consisting of three sections, were mounted onto a Superfrost Plus Microscope slide.

To examine nerve fiber diameter and myelin thickness, the ipsilateral sciatic nerve was also dissected out. Such tissues were fixed in 2% glutaraldehyde, rinsed in PBS, placed into 1% osmium tetroxide for 1 h, and finally dehydrated in increasing levels of dimethylformamide (50%, 70%, 90%, and 99%). Tissues were then embedded in reactive LR-white resin overnight. An ultramicrotome (Leica EM, UC6, Wetzlar, Germany) was used to cut transverse sections measuring 70 nm. These sections were placed on 2 × 1 mm copper grids.

### 5.5. Tissue Staining (Immunohistochemistry and Immunofluorescence)

#### 5.5.1. Immunohistochemistry (IBa1 and GFAP)

Sections were dewaxed for 20 min at 60 °C to ensure tissues adhered to the slides. Next, slides were deparaffinized by immersion in histoclear, immersed in increasingly dilute ethanol solutions (99%, 96%, and 70%), and finally rinsed in distilled water. To block endogenous peroxidase activity in the tissues, the slides were incubated in a solution of 10% H_2_O_2_ in TBS for 30 min. A citrate buffer was then used for HIER (heat-induced epitope retrieval) and subsequent antigen retrieval. Finally, the slides were washed in a buffer consisting of 0.2% milk and 0.3% Triton-X in TBS, in order to limit non-specific binding of antibodies and thereby reduce background staining. Slides were incubated overnight at 4 °C with a solution of primary antibody, that is, either polyclonal rabbit anti-IBa1 antibody (019-19741, Wako, Osaka, Japan) or polyclonal anti-GFAP antibody (z0334, Dako, Glostrup, Denmark), at a 1:1000 concentration, and TBS in 0.3% Triton-X. The following day, the slides were rinsed in TBS and then incubated at room temperature in a solution of secondary antibody (HRP-conjugated goat-anti-rabbit IgG; PO448, Dako, Glostrup, Denmark) and TBS/Triton-X buffer at a 1:400 concentration. The incubation period was 70 min and 120 min for IBa1- and GFAP-marked slides, respectively. Next, the slides were rinsed in TBS to remove any excess antibody. Secondary antibodies were detected by adding 3.3′-diaminobenzidine (DAB) dissolved in TBS and H_2_O_2_ and subsequently rinsing with slides with TBS. To generate counterstains of the nuclei, slides were submerged in Mayer’s hematoxylin for 2 min and then washed in tap water to remove excess hematoxylin. Finally, tissues were dehydrated by immersing slides into increasingly concentrated ethanol solutions (70%, 96%, 99%). The slides were mounted with coverslips using Eukitt (Freiburg, Germany) mounting medium.

#### 5.5.2. Immunofluorescence (IB4 and GABA_A_R)

Staining was conducted as described in Section 5.5.1; however, the following points were different. Firstly, the primary antibody solution consisted of either polyclonal rabbit anti-GABA (Z0334, Dako) at a 1:500 concentration or polyclonal goat-anti-Ib4 (ZF-0826, Vector Lab, Burlingame, CA, USA) at a 1:100 concentration in 1% bovine serum albumin (BSA) mixed with 0.3% Triton-X solution. The incubation period with this primary antibody solution was overnight and 3 days, respectively. Secondly, the secondary antibody solution comprised anti-rabbit 568 (835724, Invitrogen, Carlsbad, CA, USA) for GABA and anti-goat 488 (2134018, Invitrogen) for IB4, mixed with BSA/Triton-X buffer, both at a 1:400 concentration. Slides were incubated in a dark, moisturized chamber at room temperature for 2 h. Also, slides were incubated with 4′,6-diamidino-2-phenylindole (DAPI) dissolved in distilled H_2_O for 8 min, to detect secondary antibodies. Finally, fluorescence mounting medium (23023, Dako, Glostrup, Denmark) was used to mount coverslips onto these slides.

### 5.6. Stereological Analysis

#### 5.6.1. Estimating Number Density of Iba1- and GFAP-Marked Cells

A NanoZoomer 2.0 HT scanner (Hamamatsu, Japan) with a 20×/0.75 lens was used to take super images of Iba1- and GFAP-stained slides. An automated physical disector module in VIS-analysis software (Visiopharm, Hoersholm, Denmark) was used to quantify cells in these super images. Neighboring sections were aligned according to the border of the spinal cord, either manually or automatically by VIS. The computer selected fields of view (FOV) by systematic random sampling within the defined region of interest (ROI). Each FOV had a counting frame measuring 96.4 μm × 72.6 μm, and was viewed one by one, at a screen magnification of 40×. Each counting frame was paired and overlaid with the sampled images of the reference and look-up sections, thereby aligning these two sections and enabling quantification of both sections. A counting frame was only included in the count if the corner point touched spinal cord tissue. Cells had to satisfy a set of criteria that were the same for astrocytes and microglia to be included in the count. A cell was counted if the nucleus was well defined and the entire DAB-stained soma was either within the frame or touched the green inclusion line of the counting frame. Cells that touched the red exclusion line or were present in both the reference and look-up sections were not counted.

The following formula was used to determine the number density of either astrocytes or microglia:

NV=ΣQ−(cell)ap·ΣP·t·2

where the number density N_V_ is calculated as the total astrocyte or microglia count ($\Sigma Q^{-}(cell)$) by comparing the reference section and the look-up section, divided by the product of the total investigated disector volume.

#### 5.6.2. Quantifying GABA Intensity

Slides were photographed on a Leica DM6000B microscope (Wetzlar, Germany) with a CTR6000 motorized 4-slide stage (Wetzlar, Germany) and a MAC 5000 XY Stepper Motor Stage controller (Wetzlar, Germany) and joystick control unit (Ludl Electronic Products Ltd., Hawthorne, NY, USA). The Leica microscope was connected to a computer, and VIS analysis software (version 6.4.1.2240) was used to take the images. Slides were photographed under a 40× lens (Wetzlar, Germany). IB4-marked slides were photographed with an L5 filter and an exposure time of 347 ms, while GABA-marked slides were photographed with an N21 filter and an exposure time of 893 ms. NIH freeware program ImageJ (version 2.0.0) was used to analyze these images. Only cells in the dorsal horn were considered, and for each cell, four regions were outlined using a freehand drawing tool: cell body, nucleus, and two areas just beside the cell (background readings). The ROI tool of ImageJ was used to measure the area, mean gray value, integrated density, and raw integrated density of the marked regions. The left and right sides of each spinal cord section were analyzed separately. The following equation was derived from a protocol published by QBI, The University of Queensland, Australia, and was used to determine the adapted corrected total cell fluorescence (aCTCF):

*aCTCF* = *IntDen* − (*area* · *mean_NegCont_*)

where integrated density (IntDen) is the product of the area and mean gray value, area is the area of the cytoplasm, and mean_NegCont_ is the average fluorescence of the two background readings. aCTCF is reported as mean fold change (MFC), which corresponds to the aCTCF of the ipsilateral half divided by the aCTCF of the contralateral half.

#### 5.6.3. Measuring Sciatic Nerve Parameters Using Transmission Electron Microscopy (TEM)

Image acquisition was carried out using a Jeol JEM-1400 Plus transmission electron microscope (Tokyo, Japan). Systematic and uniformly random sampling of fields of view (FOVs) was conducted with the aid of SerialEM software (Version 3.7.13) [47]. Measurements of axon diameter, myelin thickness, and fiber diameter (axon and myelin diameter) were performed in the microimager module of VIS. Estimations were derived from systematic sampling of FOVs with counting frames measuring 700 μm^2^, distributed randomly and systematically 125 μm apart, combined with a 2D-nucleator technique [38].

$$Myelin\;thickness({\mu m}^{2})=\frac{r_{m}-r}{2}$$

where r_m_ is defined as the axon diameter including the myelin sheath, and r is defined as the axon diameter excluding the myelin sheath.

Type A fibers were defined as large, with a myelin sheath. Type B fibers were small, with a myelin sheath. C-fibers were the smallest, with no myelin [48].

### 5.7. Statistical Analysis

All data derived from tests and observations are expressed as the mean ± SD, unless stated otherwise. The body weight data of the mice were analyzed using Student’s *t*-test, while von Frey data were analyzed using mixed-design ANOVA, and other data were analyzed using one-way ANOVA with a post hoc test where appropriate. The analysis was performed in GraphPad Prism v9, and the significance level was set to *p* < 0.05.

## Data Availability

The datasets presented in this article are not readily available because the data are part of an ongoing study. Requests to access the datasets should be directed to jrnyengaard@clin.au.dk.
